# Prenatal smoking exposure and psychiatric symptoms in adolescence

**DOI:** 10.1111/j.1651-2227.2006.00148.x

**Published:** 2007-03-01

**Authors:** Marit S Indredavik, Ann-Mari Brubakk, Pål Romundstad, Torstein Vik

**Affiliations:** 1 Department of Neuroscience, Norwegian University of Science and Technology Trondheim, Norway and St. Olavs Hospital Trondheim, Norway; 2 Department of Laboratory Medicine, Children's and Women's Health, Norwegian University of Science and Technology Trondheim, Norway; 3 Department of Public Health and General Practice, Norwegian University of Science and Technology Trondheim, Norway

**Keywords:** Adolescent, Mental health, Pregnancy, Tobacco smoke

## Abstract

**Aim:**

Explore associations between smoking in pregnancy and psychiatric symptoms in the adolescent offspring.

**Design/subjects:**

A prospective population based follow-up of 84 adolescents at 14 years of age, of whom 32 of the mothers reported smoking during pregnancy.

**Main outcome measures:**

The Achenbach System of Empirically Based Assessment (ASEBA), ADHD-Rating Scale IV, Autism Spectrum Screening Questionnaire (ASSQ), Children's Global Assessment Scale (CGAS), estimated IQ based on four subscales of WISC-III.

**Results:**

Adolescents who were born by smokers had significantly more rule-breaking and aggressive behaviour, externalizing and total problems on the ASEBA than adolescents of non-smokers (p < 0.01), when reported by mothers, fathers and teachers. ADHD symptoms were reported more frequently (p < 0.05), and mothers also reported more internalizing symptoms (p < 0.05) and social problems (p < 0.001). The ASSQ sum score was higher (p < 0.001), and overall function as measured by the CGAS was lower (p < 0.01) for the smoking-exposed group. Associations were still present after controlling for possible confounding factors.

**Conclusion:**

Adolescents exposed to prenatal smoking had higher scores for both externalizing and internalizing psychiatric symptoms, which could not be explained by a broad range of possible psychosocial confounders. Thus, smoking in pregnancy may be a marker for increased risk of psychiatric symptoms in the offspring.

## INTRODUCTION

Smoking during pregnancy is well known to have negative effects on the growth and development of the unborn child (1). Children who were exposed to smoking *in utero* may show cognitive deficits such as delayed language development, difficulties in learning and memory tasks, reading and mathematics, and decreased general cognitive functioning (2,3). Externalizing symptoms, especially rule-breaking and aggressive behaviour have been reported from early childhood throughout adolescence with increased risk of conduct disorder and delinquency (4–7). Associations have been reported between prenatal smoking exposure and symptoms of ADHD, although these results are less consistent (7–9). Reports on internalizing symptoms such as anxiety and depression are fewer. These studies suggest a weak association with prenatal smoking exposure which, however, may be confounded by psychosocial factors (4,5). There is one report on increased risk of autism associated with smoking exposure *in utero* (10). In most studies, smoking during pregnancy is measured retrospectively, and information on psychiatric symptoms in the offspring is often collected from the mothers only.

In this study, we wanted to explore associations between smoking in pregnancy and psychiatric symptoms in the adolescent offspring in a prospective design, using a broad assessment of psychiatric symptoms by several informants, and with a wide survey of possible confounders.

## SUBJECTS AND METHODS

### Study design

The study is a population-based follow-up of 84 adolescents at 14 years of age. The adolescents were born to mothers (para 1 and 2) randomly selected in the Trondheim part of a multicentre study between January 1986 and March 1988. Details of the multi centre study have been published elsewhere (11). At enrolment before week 20 of pregnancy, the pregnant woman was asked if she smoked cigarettes on a daily basis, and if yes, she was asked to indicate the number of cigarettes smoked per day.

The present study was carried out between November 2000 and October 2002, and included a psychiatric assessment and an evaluation of cognitive abilities.

### Study population

#### Participants

In the multi centre study there were 5722 eligible women with a singleton pregnancy. At enrolment a 10% random sample of 561 women were selected for follow up. A total of 521 delivered at term, and 126 of these belonged to the Trondheim part (1200 eligible women) of the study. In the follow up assessment at 14.2 years, 84 (67%) participated (34 boys, 50 girls). Mean birth weight was 3608 g (SD: 491) and mean gestational age 39.6 weeks (SD: 1.2). Of the 84 participants, 32 (38%) were exposed to smoking *in utero*.

#### Non-participants

There were no differences in mothers' age at childbirth, duration of pregnancy, or the infants' birth weight between those who participated and those who did not consent in the two groups (data not shown).

### Methods

#### Psychiatric symptoms

We used the Achenbach System of Empirically Based Assessment (ASEBA) (12), with Youth Self Report (YSR), Child Behaviour Check List (CBCL) age 4–18 years, rated separately by mothers and fathers, and Teacher Report Form (TRF). The ASEBA is a screening instrument on emotional and behavioural symptoms with 105 (YSR) and 120 (CBCL/TRF) problem items (rated 0–1–2). The items constitute eight syndrome scores: Withdrawn, Somatic Complaints, Anxious/Depressed, Social Problems, Thought Problems, Attention Problems, Rule-Breaking Behaviour and Aggressive Behaviour. Three composite scales are computed: Internalizing Scale (Withdrawn, Somatic Complaints and Anxious/Depressed), Externalizing Scale (Rule-Breaking and Aggressive Behaviour) and Total Problems (all syndrome scores).

ADHD-Rating Scale IV (13) was rated by mothers, fathers and teachers. The scale consists of 18 questions rated 0–1–2, constituting two subscales (inattention and hyperactivity) which are added to a total score. Autism Spectrum Screening Questionnaire (ASSQ) (14), with 27 items rated 0–1–2, and Children's Global Assessment Scale (CGAS) (15), measuring the child's overall functioning from zero (low functioning) to 100 (high functioning), were rated by a child psychiatrist based on separate interviews with the adolescent and the parents.

#### Cognitive abilities

An estimate of intelligence quotient (IQ_est_) was calculated using four of Wechsler Intelligence Scales (WISC-III) (16); Vocabulary, Arithmetic, Block design and Picture arrangement.

#### Parents' mental health and socioeconomic status

Mothers (and fathers in a sub sample) completed Symptom Checklist-90-R (SCL-90-R) (17), and we used the Global Severity Index as a summary measure of psychological distress. Socioeconomic status (SES) was calculated according to Hollingshead's Two Factor Index of Social Position, based on a combination of parents' education and occupation (18).

### Ethics

The Regional Committee for Medical Research Ethics approved the study protocol (reference number 78-00; May 29, 2000). Written informed consent was obtained from both adolescents and parents.

### Statistical analysis

SPSS version 13.0, SPSS Inc., Chicago, USA was used for data analysis. In consistence with the ASEBA manual (12), and in order to compare our results with other studies using this instrument, we have presented the ASEBA raw score results as mean values with standard deviations, even though data were not strictly normally distributed. Two-group comparisons were made using Chi-Square test for dichotomous variables, Mann–Whitney *U*-test for ordinal variables, and independent samples *t*-test for continuous variables. We used general linear modelling (GLM) to adjust for potential confounding. The dependent variables were ln transformed to obtain satisfactory normal distribution of the residuals, and thus the resulting adjusted means are geometric means. A two-sided p-value < 0.05 was considered statistical significant.

## RESULTS

### Group characteristics

All children were born at term (Table S1). The proportion of boys and girls in each group did not differ. Mean number of cigarettes pr. day in the smoker group was 10.0 (SD 4.8). Of the 32 adolescents exposed to smoking *in utero*, 6 (19%) were living in single parent families at follow-up, compared with 5 of 52 (10%) in the unexposed group (ns). Birth weight was lower in the group exposed to smoking in pregnancy compared with the unexposed group. There were no group-differences in the mothers' mental health or their present alcohol consume. Socioeconomic status was lower and the mothers were younger in the smoker-group compared with the non-smoker group.

### Associations between smoking exposure in utero and psychiatric symptoms

On ASEBA, the youth self-reports indicated no group differences in the internalizing, externalizing or total problems composite scales (Table S2). However, smoking exposed adolescents reported higher scores on the subscales thought (p = 0.03) and attention problems (p = 0.05) than the unexposed group. Mothers, fathers and teachers consistently reported more rule-breaking and aggressive behaviour, externalizing and total problems in the smoking exposed compared with the unexposed group (p < 0.01). Mothers reported higher scores on the social problems scale (p < 0.001), so did fathers and teachers (p ≤ 0.05). Mothers also reported more internalizing symptoms, which reflected higher scores on the withdrawn and anxious/depressed subscales (p ≤ 0.05). Fathers reported higher scores on the thought problems scale (p < 0.01).

Increased scores for ADHD symptoms, both inattention and hyperactivity, was reported by teachers on ASEBA (p ≤ 0.01) (Table S2), and by mothers, fathers and teachers on the ADHD-Rating Scale IV (p < 0.05) (Table S3) for the exposed group compared with the unexposed group.

The sum score on the Autism Spectrum Screening Questionnaire was higher for the exposed group than for the unexposed group (p < 0.001) (Table S3). In addition, mean IQ_est_ was 10.4 (95% CI: 3.1–17.7) points lower in the smoking-exposed group. Overall functioning, as measured by the CGAS, was lower for the exposed group (p < 0.01), however, the score was within the normal range (above 80).

When analyses were done separately for boys and girls, the main differences between the exposed and the non-exposed groups persisted (data not shown).

### Multivariable analyses

General linear modelling was used to control for possible confounding factors when investigating the associations between smoking exposure *in utero* and psychiatric symptoms as reported by mothers (Table S4). When we controlled for gender, birth weight, socioeconomic status, maternal age, single parent, mothers' mental health and mothers' present use of alcohol, a slight attenuation of the association was observed, however, exposure to smoking *in utero* was still associated with higher symptom scores on the ASSQ, the ASEBA externalizing scale and total problems (p < 0.01), and higher scores on ADHD-Rating Scale IV and ASEBA internalizing scale (p < 0.05).

We found no evidence for interaction between gender and smoking (p-values ranging from 0.32 to 0.99).

Fathers' mental health was recorded in 66 adolescents (25 exposed and 41 unexposed). When we adjusted also for fathers' mental health in this sub sample, the effects of smoking were slightly attenuated (8–15%), however, the main results were still unchanged.

## DISCUSSION

In this study, we have found higher psychiatric symptom scores in adolescents who were exposed to smoking *in utero* compared with unexposed adolescents at 14 years of age. Adolescents who were born by smokers had significantly more rule-breaking and aggressive behaviour, symptoms of ADHD, social problems and higher sum score on the ASSQ than adolescents of non-smokers. Furthermore, more internalizing symptoms such as withdrawal and anxious/depressed symptoms were reported by mothers. Smoking-exposure *in utero* was also associated with lowered overall functioning.

### Strengths and limitations of the study

Strengths of the present study are firstly its prospective design with information on smoking collected during pregnancy. Secondly, psychiatric symptoms were broadly assessed with several instruments reported by the adolescents themselves, their mothers, fathers and teachers. Possible confounding factors were also widely surveyed.

The sample size was moderate, resulting in low power to demonstrate small differences. Hence, non-significant findings should be interpreted with caution. Yet, significant differences were found between the exposed and the unexposed group. Furthermore, the main outcomes for smoking-exposed adolescents were consistently reported by mothers, fathers, and teachers, and across several instruments. Therefore, it is unlikely that these results are due to chance.

Information on smoking during pregnancy was collected during the second trimester, hence, any recall bias can be ruled out. Birth weight did not differ between participants and those who did not consent to participation, which may indicate that the main results are less likely due to selection bias.

Parents of tobacco-exposed adolescents might know that their child could be at increased risk of health problems, and their information could be biased. Knowledge on the risk of tobacco-smoking could, on the other hand, lead to less reported problems. But in this study, the hypothesis was unknown for the informants as well as the investigator. Furthermore, the teachers were presumably unaware of the mothers` smoking during pregnancy. Their reports corresponded with the parents' reports and may suggest that the main results were not caused by information bias.

The adolescents themselves reported fewer problems than did their parents, which is consistent with other studies regarding externalizing behaviour (19). Mothers reported more internalizing problems among tobacco exposed than among non-exposed adolescents, whereas the differences between the two groups reported by fathers and teachers were not statistically significant. The latter is most likely due to a lower number of respondents among fathers and teachers than among mothers, since all three groups of respondents reported nearly identical mean scores for internalizing problems.

After adjusting for possible confounders such as gender, birth weight, socioeconomic status, single parent, mothers' present use of alcohol, mothers' age and mothers' mental health, the increased risk of psychiatric symptoms among tobacco exposed adolescents persisted, although slightly attenuated.

### Characteristics of the tobacco-exposed adolescents

The results support and supplement the literature on the association between maternal smoking during pregnancy and mental health problems in the offspring. Our finding of higher symptom rates for rule-breaking and aggressive behaviour in the exposed group is consistent with a large amount of research (4–7). Furthermore, teachers reported significantly higher scores on both inattention and hyperactivity/impulsivity on the ASEBA specific scales for ADHD symptoms. Even the adolescents themselves reported attention problems, and we found increased ADHD symptom scores on mother report after controlling for a range of possible confounding variables. Previous studies have reported similar findings (9). Rodriguez and Bohlin found ADHD symptoms at 7 years to be associated with maternal smoking and stress during pregnancy, particularly for boys (20). Kotimaa et al. studied over 9000 children at 8 years of age, and found a positive dose-respons relationship between maternal smoking during pregnancy and hyperactivity (8). There is, however, a discussion as to whether there is an increased risk of ADHD per se as Linnet et al. have reported, or if this risk is linked to the presence of comorbid oppositional defiant disorder as described by Wakschlag et al. (7,9). In a twin study by Button et al. both antisocial behaviour and ADHD symptoms were independently influenced by maternal prenatal smoking during pregnancy (6).

Our study includes several measures of social functioning; the social problems subscale on ASEBA scored by different informants and the ASSQ sum score. Mothers reported higher social problems score on ASEBA for the smoking exposed adolescents, supported by weaker associations reported by fathers and teachers. This is consistent with other studies; in a large Australian cohort of children at 5 years of age, Williams et al. found a relationship between smoking-exposure during pregnancy and social problems (5). Furthermore, the ASSQ sum score was strongly associated with smoking exposure in our study. This score may express social problems, or more specifically, the score is in fact a measure of social sensitivity, as the instrument is designed to screen symptoms of Asperger syndrome and other high functioning autism spectrum disorders (14). Hence, the combination of measures in our study indicates that social problems are not only secondary to conduct problems, but may reflect reduced sensitivity in social interaction. No association was found between smoking in pregnancy and autistic disorders, as reported by Hultman et al. (10). This may be due to the limited size of our study.

Mothers reported more internalizing symptoms for the exposed group compared with the unexposed group. This was mainly present for the withdrawn and the anxious/depressed subscales. Williams et al. reported a weak relationship between maternal smoking during pregnancy and internalizing symptoms at 5 years of age (5). In 16–18 year olds, Fergusson et al. found increased rates of depression, however, not after controlling for confounding variables, including family and social background factors (4). In contrast, we found that the increased rate of internalizing symptoms was still present after controlling for psychosocial factors. Hence, our results point to a valid association between smoking exposure and internalizing symptoms.

Decreased cognitive functioning and deficits in learning and memory tasks are reported to be related to maternal smoking during pregnancy (3). In a review, Weitzman et al. conclude that prenatal smoking is associated with a modest decrement of 4–5 IQ points, and lowered school performance (21). We found a decrement of 10 IQ points in the smoking exposed group, which falls in line with the previous literature, although the difference in our study is noticable larger. This may be caused by the estimation of IQ based on four subscales (vocabulary, arithmetic, block design, and picture arrangement), which is a crude approximation. In fact, the use of the arithmetic subscale may have influenced the estimated total score in a negative direction, as performance in mathematics is reported to be specifically affected by prenatal smoking (2).

The CGAS score is a non-specific score for overall functioning in relation to mental health problems (15). We are not aware of other studies on prenatal smoking exposure using this measure, designed to show the impact of psychiatric symptoms. Even though the CGAS score in the smoking exposed group was within the range of normal functioning (above 80), the score was significantly lower than in the unexposed group. This illustrates that the adolescents not only had psychiatric symptoms compared with the unexposed group, but that these symptoms were affecting their every day functioning.

The results were mainly the same for boys and girls. In the general population, externalizing behaviour is more frequent for boys, whereas girls are more prone to develop internalizing symptoms (22). It is thus noteworthy that associations between smoking-exposure and externalizing behaviour in fact were also demonstrated for girls. In a review, associations between prenatal smoking and externalizing behaviour are described to be more consistent for boys than for girls (23). However, other studies have reported parallel findings for boys and girls in childhood and adolescence, in conformity with our study (24,25).

### Associations and causal relationship

Although a valid association cannot prove a causal relationship, we may discuss whether a biological mechanism is plausible. A toxic effect on the fetus' developing nervous system is possible through carbon monoxide and nicotine as the main toxic components of cigarette smoking, leading to dysregulation in neurodevelopment (26). These results are supported by animal studies (27). A dose-response relationship has also been reported, consistent with a causal relationship (8).

Genetics may contribute with heredity factors associated with both smoking and externalizing behaviour (23). A twin study concluded with a genetic influence in ADHD, however, with an additive effect of smoking in pregnancy (28). Genes and chromosomes may also interact with smoking, increasing the vulnerability for mental disorders (29,30). Further neurobiological research is needed to establish a potential causal mechanism.

Irrespective of causal pathways, smoking in pregnancy seems to be a marker for increased risk of psychiatric symptoms in the offspring. As long as a direct toxic effect is possible, and a proof to the contrary is lacking, there is every reason to be cautious. Awaiting further evidence, smoking pregnant women must not only be advised to stop smoking, but should be treated as a potential risk group regarding their children's mental health.

## CONCLUSION

We found that adolescents born by mothers who smoked during pregnancy had higher symptom scores for rule-breaking and aggressive behaviour, symptoms of ADHD, social problems, and also internalizing symptoms. In addition, smoking-exposure was associated with lowered overall functioning. The psychiatric results could not be explained by the range of psychosocial factors that were evaluated. Thus, smoking in pregnancy may be a marker for an increased risk of psychiatric symptoms in the offspring.

## Supplementary material

The following supplementary material is available for this article:

Table S1 Group characteristics in adolescents according to exposure for tobacco-smoking *in utero*

Table S2 Raw scores on Achenbach System of Empirically Based Assessment (ASEBA) in adolescents according to exposure for tobacco-smoking *in utero*

Table S3 Psychiatric symptoms and estimated intelligence quotient in adolescents according to exposure for tobaccosmoking *in utero*

Table S4 Unadjusted and adjusted analysis of mental health in adolescents according to exposure for tobacco-smoking *in utero*

This material is available as part of the online article from: http://www.blackwell-synergy.com/doi/abs/10.1111/j.1651-2227.2006.00148.x (This link will take you to the article abstract).

## References

[1] Hofhuis W, de Jongste JC, Merkus PJ (2003). Adverse health effects of prenatal and postnatal tobacco smoke exposure on children. Arch Dis Child.

[2] Batstra L, Hadders-Algra M, Neeleman J (2003). Effect of antenatal exposure to maternal smoking on behavioural problems and academic achievement in childhood: prospective evidence from a Dutch birth cohort. Early Hum Dev.

[3] Huizink AC, Mulder EJ (2006). Maternal smoking, drinking or cannabis use during pregnancy and neurobehavioral and cognitive functioning in human offspring. Neurosci Biobehav Rev.

[4] Fergusson DM, Woodward LJ, Horwood LJ (1998). Maternal smoking during pregnancy and psychiatric adjustment in late adolescence. Arch Gen Psychiatry.

[5] Williams GM, O'Callaghan M, Najman JM, Bor W, Andersen MJ, Richards D (1998). Maternal cigarette smoking and child psychiatric morbidity: a longitudinal study. Pediatrics.

[6] Button TM, Thapar A, McGuffin P (2005). Relationship between antisocial behaviour, attention-deficit hyperactivity disorder and maternal prenatal smoking. Br J Psychiatry.

[7] Wakschlag LS, Pickett KE, Kasza KE, Loeber R (2006). Is prenatal smoking associated with a developmental pattern of conduct problems in young boys?. J Am Acad Child Adolesc Psychiatry.

[8] Kotimaa AJ, Moilanen I, Taanila A, Ebeling H, Smalley SL, McGough JJ (2003). Maternal smoking and hyperactivity in 8-year-old children. J Am Acad Child Adolesc Psychiatry.

[9] Linnet KM, Wisborg K, Obel C, Secher NJ, Thomsen PH, Agerbo E (2005). Smoking during pregnancy and the risk for hyperkinetic disorder in offspring. Pediatrics.

[10] Hultman CM, Sparén P, Cnattingius S (2002). Perinatal risk factors for infantile autism. Epidemiology.

[11] Bakketeig LS, Jacobsen G, Hoffman HJ, Lindmark G, Bergsjø P, Molne K (1993). Pre-pregnancy risk factors of small-for-gestational age births among parous women in Scandinavia. Acta Obstet Gynecol Scand.

[12] Achenbach TM, Rescorla LA (2001). Manual for the ASEBA School-Age Forms & Profiles.

[13] Barkley RA, Murphy KR (1998). Attention-deficit hyperactivity disorder: a clinical workbook.

[14] Ehlers S, Gillberg C, Wing L (1999). A screening questionnaire for Asperger syndrom and other high-functioning autism spectrum disorders in school age children. J Autism Dev Disord.

[15] Shaffer D, Gould MS, Brasic J, Ambrosini P, Fisher P, Bird H (1985). A Children's Global Assessment Scale (CGAS) (for children 4 to 16 years of age). Psychopharmacol Bull.

[16] Wechsler D (1999). Wechsler Intelligence Scale for Children.

[17] Derogatis LR (1994). Symptom Checklist-90-R. Administration, scoring, and procedures manual.

[18] Hollingshead AB (1958). Two factor index of social position.

[19] Handwerk ML, Larzelere RE, Soper SH, Friman PC (1999). Parent and child discrepancies in reporting severity of problem behaviors in three out-of-home settings. Psychol Assess.

[20] Rodriguez A, Bohlin G (2005). Are maternal smoking and stress during pregnancy related to ADHD symptoms in children?. J Child Psychol Psychiatry.

[21] Weitzman M, Byrd RS, Aligne CA, Moss M (2002). The effects of tobacco exposure on children's behavioral and cognitive functioning: implications for clinical and public health policy and future research. Neurotoxicol Teratol.

[22] Graham P, Turk J, Verhulst F, Graham P, Turk J, Verhulst F (1999). Classification and prevalence of psychiatric disorders. Child psychiatry: a developmental approach.

[23] Wakschlag LS, Pickett KE, Cook E, Benowitz NL, Leventhal BL (2002). Maternal smoking during pregnancy and severe antisocial behavior in offspring: a review. Am J Public Health.

[24] Höök B, Cederblad M, Berg R (2006). Prenatal and postnatal maternal smoking as risk factors for preschool children's mental health. Acta Paediatr.

[25] Weissman MM, Warner V, Wickramaratne PJ, Kandel D (1999). Maternal smoking during pregnancy and psychopathology in offspring followed to adulthood. J Am Acad Child Adolesc Psychiatry.

[26] Ernst M, Moolchan ET, Robinson ML (2001). Behavioral and neural consequences of prenatal exposure to nicotine. J Am Acad Child Adolesc Psychiatry.

[27] Pauly JR, Sparks JA, Hauser KF, Pauly TH (2004). In utero nicotine exposure causes persistent, gender-dependant changes in locomotor activity and sensitivity to nicotine in C57Bl/6 mice. Int J Dev Neurosci.

[28] Thapar A, Fowler T, Rice F, Scourfield J, van den Bree M, Thomas H (2003). Maternal smoking during pregnancy and attention deficit hyperactivity disorder symptoms in offspring. Am J Psychiatry.

[29] Kahn RS, Khoury J, Nichols WC, Lanphear BP (2003). Role of dopamine transporter genotype and maternal prenatal smoking in childhood hyperactive-impulsive, inattentive, and oppositional behaviors. J Pediatr.

[30] de la Chica RA, Ribas I, Giraldo J, Egozcue J, Fuster C (2005). Chromosomal instability in amniocytes from fetuses of mothers who smoke. JAMA.

