# Supplementary material for: Prenatal smoking exposure and psychiatric symptoms in adolescence
Source: Acta Paediatr. 2007 Mar 1;96(3):377–82. doi: 10.1111/j.1651-2227.2006.00148.x (PMC2049061; doi:10.1111/j.1651-2227.2006.00148.x)
Supplement: Table S1 — Group characteristics in adolescents according to exposure for tobacco-smoking in utero [file apa0096-0377-ts1.doc]

**Table S1** Group characteristics in adolescents according to exposure for tobacco-smoking *in utero*

|  | **Smoker group** | | **Non-smoker group** | |  |
| --- | --- | --- | --- | --- | --- |
|  | Mean | (SD) | Mean | (SD) |  |
|  | n = 32 | | n = 52 | | p-value^*^ |
|  |  |  |  |  |  |
| Mothers’ age at childbirth | 28.9 | (4.3) | 31.0 | (4.1) | 0.03 ^b^ |
| Cigarettes per day mothers ^†^ | 10.0 | (4.8) | 0 | (0) | <0.001 ^b^ |
| Present socioeconomic status | 3.5 | (1.1) | 4.2 | (0.9) | 0.004 ^a^ |
| Mothers’ present mental health ^‡^ | 0.28 | (0.22) | 0.26 | (0.23) | 0.6 ^a^ |
| Mothers’ present use of alcohol | 2.0 | (0.8) | 2.0 | (0.8) | 1.0 ^a^ |
|  |  |  |  |  |  |
| Birth weight | 3440 | (480) | 3712 | (473) | 0.01 ^b^ |
| Gestational age | 39.5 | (1.3) | 39.6 | (1.1) | 0.8 ^b^ |
| Age of adolescents | 14.2 | (0.4) | 14.2 | (0.3) | 1.0 ^b^ |
|  |  |  |  |  |  |
|  |  |  |  |  |  |
|  | Number | (%) | Number | (%) | p-value^*^ |
| Boys | 13 | (41) | 21 | (40) | 1.0 ^c^ |
| Girls | 19 | (59) | 31 | (60) | 1.0 ^c^ |
| Single parent | 6 | (19) | 5 | (10) | 0.2 ^c^ |
|  |  |  |  |  |  |

^*^ p-value denotes difference between smoker and non-smoker group

^†^ Number of cigarettes per day at time of conception

^‡^ Symptom Checklist-90-Revised. Global symptom index [17]

Two-group comparisons with Mann Whitney U-test ^a^, independent samples t-test ^b^ and Chi-square test ^c^ (dichotomous variables)

**Table S2** Raw scores on Achenbach System of Empirically Based Assessment (ASEBA) in adolescents according to exposure for tobacco-smoking *in utero*

|  | **Exposed** | | **Unexposed** | | |  |
| --- | --- | --- | --- | --- | --- | --- |
|  | Mean | (SD) | Mean | | (SD) |  |
|  | n = 32 | | n = 52 | | | p-value^*^ |
| **ASEBA Self-report (YSR)** (n=32/52) |  |  |  |  | |  |
|  |  |  |  | |  |
| Withdrawn | 1.8 | (1.4) | 2.2 | (1.9) | | 0.7 |
| Somatic Complaints | 2.3 | (2.8) | 1.8 | (1.8) | | 0.8 |
| Anxious/Depressed | 3.7 | (3.7) | 3.1 | (3.7) | | 0.4 |
| Social Problems | 1.5 | (1.6) | 1.4 | (1.5) | | 0.9 |
| Thought Problems | 1.8 | (1.9) | 0.9 | (1.4) | | 0.03 |
| Attention Problems | 4.0 | (2.7) | 2.8 | (2.5) | | 0.05 |
| Rule-Breaking Behaviour | 2.8 | (2.7) | 1.9 | (1.8) | | 0.2 |
| Aggressive Behaviour | 6.8 | (5.4) | 5.5 | (4.0) | | 0.4 |
| **Internalizing Scale** | 7.6 | (6.3) | 6.9 | (6.0) | | 0.6 |
| **Externalizing Scale** | 9.6 | (7.6) | 7.4 | (5.3) | | 0.3 |
| **Total Problems** | 29.0 | (19.0) | 22.5 | (14.4) | | 0.2 |
| **ASEBA Mother-report (CBCL)** (n=32/52) |  |  |  |  | |  |
|  |  |  |  | |  |
| Withdrawn | 1.2 | (1.2) | 0.8 | (1.5) | | 0.03 |
| Somatic Complaints | 1.5 | (1.9) | 1.0 | (1.4) | | 0.1 |
| Anxious/Depressed | 1.6 | (2.3) | 1.0 | (1.6) | | 0.05 |
| Social Problems | 0.8 | (0.8) | 0.3 | (0.7) | | <0.001 |
| Thought Problems | 0.1 | (0.4) | 0.0 | (0.0) | | 0.2 |
| Attention Problems | 1.8 | (1.9) | 1.0 | (1.4) | | 0.02 |
| Rule-Breaking Behaviour | 1.1 | (1.7) | 0.2 | (0.4) | | <0.001 |
| Aggressive Behaviour | 3.4 | (3.4) | 1.6 | (1.9) | | 0.005 |
| **Internalizing Scale** | 4.2 | (4.1) | 2.7 | (3.1) | | 0.02 |
| **Externalizing Scale** | 4.5 | (4.5) | 1.8 | (2.1) | | <0.001 |
| **Total Problems** | 13.1 | (10.3) | 6.8 | (6.6) | | 0.001 |
| **ASEBA Father-report (CBCL)** (n=25/42) |  |  |  |  | |  |
|  |  |  |  | |  |
| Withdrawn | 1.0 | (1.5) | 0.6 | (1.1) | | 0.3 |
| Somatic Complaints | 1.5 | (2.2) | 1.0 | (1.1) | | 0.6 |
| Anxious/Depressed | 1.8 | (2.4) | 0.9 | (1.5) | | 0.07 |
| Social Problems | 0.8 | (1.2) | 0.3 | (0.6) | | 0.03 |
| Thought Problems | 0.2 | (0.5) | 0.0 | (0.0) | | 0.008 |
| Attention Problems | 1.8 | (2.4) | 0.8 | (1.2) | | 0.1 |
| Rule-Breaking Behaviour | 1.5 | (2.5) | 0.1 | (0.3) | | <0.001 |
| Aggressive Behaviour | 3.6 | (4.3) | 1.1 | (1.4) | | 0.004 |
| **Internalizing Scale** | 4.2 | (5.3) | 2.4 | (2.5) | | 0.2 |
| **Externalizing Scale** | 5.1 | (6.3) | 1.2 | (1.5) | | 0.001 |
| **Total Problems** | 14.0 | (15.0) | 5.3 | (4.6) | | 0.006 |
| **ASEBA Teacher-report (TRF)** (n=29/44) |  |  |  |  | |  |
|  |  |  |  | |  |
| Withdrawn | 1.1 | (1.8) | 0.4 | (0.7) | | 0.06 |
| Somatic Complaints | 0.6 | (1.5) | 0.4 | (0.7) | | 0.9 |
| Anxious/Depressed | 2.6 | (3.4) | 1.3 | (1.7) | | 0.1 |
| Social Problems | 1.9 | (2.7) | 0.6 | (1.3) | | 0.02 |
| Thought Problems | 0.1 | (0.3) | 0.0 | (0.2) | | 0.3 |
| Attention Problems | 7.6 | (9.0) | 2.8 | (5.1) | | 0.009 |
| Rule-Breaking Behaviour | 1.7 | (2.6) | 0.4 | (1.2) | | 0.002 |
| Aggressive Behaviour | 6.7 | (8.8) | 1.2 | (2.8) | | 0.004 |
| **Internalizing Scale** | 4.2 | (5.8) | 2.0 | (2.2) | | 0.2 |
| **Externalizing Scale** | 8.4 | (10.9) | 1.6 | (3.9) | | 0.006 |
| **Total Problems** | 22.0 | (24.5) | 7.1 | (10.7) | | 0.005 |
| Inattention | 5.6 | (6.6) | 2.3 | (4.0) | | 0.01 |
| Hyperactivity-Impulsivity | 4.2 | (5.0) | 1.3 | (2.6) | | 0.007 |

^*^p-value denotes difference between smoking-exposed and not smoking-exposed adolescents

Achenbach System of Empirically Based Assessment (ASEBA) [12]

Two-group comparisons with Mann Whitney U-test

**Table S3** Psychiatric symptoms and estimated intelligence quotient in adolescents according to exposure for tobacco-smoking *in utero*

|  | **Exposed** | | **Unexposed** | |  |
| --- | --- | --- | --- | --- | --- |
|  | Mean | (SD) | Mean | (SD) |  |
|  | n = 32 | | n = 52 | | p-value^*^ |
| **Sum score ASSQ**^†^ (n=30/47) | 3.2 | (2.6) | 1.5 | (2.4) | <0.001 ^a^ |
| **ADHD-Rating Scale IV**^‡^ |  |  |  |  |  |
|  |  |  |  |  |
| **Mother report:** (n=32/52) |  |  |  |  |  |
| Inattention | 4.4 | (4.7) | 2.3 | (2.7) | 0.009 ^a^ |
| Hyperactivity | 2.7 | (2.7) | 1.1 | (1.5) | 0.005 ^a^ |
| Total | 7.2 | (6.9) | 3.4 | (3.9) | 0.003 ^a^ |
| **Father report:** (n=26/44) |  |  |  |  |  |
| Inattention | 3.8 | (3.2) | 2.2 | (2.9) | 0.02 ^a^ |
| Hyperactivity | 2.5 | (3.0) | 0.6 | (1.0) | <0.001 ^a^ |
| Total | 6.2 | (5.6) | 2.8 | (3.3) | 0.003 ^a^ |
| **Teacher report:** (n=28/44) |  |  |  |  |  |
| Inattention | 5.9 | (7.4) | 2.4 | (4.3) | 0.03 ^a^ |
| Hyperactivity | 4.2 | (6.2) | 1.1 | (2.5) | 0.01 ^a^ |
| Total | 10.1 | (13.3) | 3.6 | (6.4) | 0.02 ^a^ |
|  |  |  |  |  |  |
| **CGAS**^§^ (n=30/47) | 81.1 | (8.6) | 86.8 | (7.9) | 0.004 ^b^ |
| **Estimated Intelligence Quotient (IQ)** (n=30/47) |  |  |  |  |  |
|  |  |  |  |  |
| Estimated verbal IQ | 86.6 | (13.7) | 95.5 | (15.3) | 0.01 ^b^ |
| Estimated performance IQ | 93.1 | (21.0) | 102.9 | (16.9) | 0.03 ^b^ |
| Estimated full scale IQ | 88.2 | (17.2) | 98.6 | (14.7) | 0.006 ^b^ |
|  |  |  |  |  |  |

^*^p-value denotes difference between smoking-exposed and not smoking-exposed adolescents

^†^Autism Spectrum Screening Questionnaire [14]

^‡^Attention Deficit/Hyperactivity Disorder-Rating Scale IV [13]

^§^Children’s Global Assessment Scale [15]

Two-group comparisons with Mann Whitney U-test ^a^ and independent samples t-test ^b^

**Table S4** Unadjusted and adjusted analysis of mental health in adolescents according to exposure for tobacco-smoking *in utero*

|  |  | **Unadjusted** | | | | | | | **Adjusted^$^** | | | | | | |
| --- | --- | --- | --- | --- | --- | --- | --- | --- | --- | --- | --- | --- | --- | --- | --- |
|  |  | Mean^†^ | | (95% CI ) | | p-value | | | Mean^†^ | | (95% CI) | | p-value^*^ | | |
| **ADHD rating scale**^‡^ | |  | |  | |  | | |  | |  | | |  | |
| Total score | Exposed (32) | 5.80 | | (4.29 – 7.85) | |  | | | 4.93 | | (3.14 – 7.74) | | |  | |
|  | Unexposed (52) | | 3.09 | (2.43 – 3.91) | | 0.002 | | | 3.20 | | (1.99 – 5.14) | | | 0.04 | |
|  | | | | | | | | | | | | | | | |
| **ASEBA**^§^ |  | | | | | | | | | | | | | | |
| Internalizing scale | Exposed (32) | 4.14 | | | (3.17 – 5.42) | |  | | | 3.92 | | (2.65 – 5.79) | |  | |
|  | Unexposed (52) | 2.66 | | | (2.15 – 3.28) | | 0.01 | | | 2.65 | | (1.75 – 3.99) | | 0.04 | |
|  |  | | | | | | | | | | | | | | |
| Externalizing scale | Exposed (32) | 4.05 | | | (3.09 – 5.31) | |  | | | 4.19 | | (2.82 – 6.22) | | |  |
|  | Unexposed (52) | 2.09 | | | (1.69 – 2.59) | | <0.001 | | | 2.38 | | (1.57 – 3.61) | | | 0.003 |
|  |  | | | | | | | | | | | | | | |
| Total score | Exposed (32) | 11.01 | | | (8.22 – 14.76) | | |  | | 10.59 | | (7.08 – 15.84) | |  | |
|  | Unexposed (52) | 5.58 | | | (4.43 – 7.01) | | | <0.001 | | 5.74 | | (3.76 – 8.77) | | 0.002 | |
|  | | | | | | | | | | | | | | | |
| **ASSQ**^¶^ |  | | | | | | | | | | | | | | |
| Sum score | Exposed (30) | 3.43 | | | (2.67 – 4.39) | | |  | | 3.26 | | (2.21 – 4.79) | | |  |
|  | Unexposed (47) | 1.87 | | | (1.54 – 2.28) | | | <0.001 | | 1.87 | | (1.25 – 2.78) | | | 0.003 |
|  | | | | | | | | | | | | | | | |

^*^p-value denotes difference between smoking-exposed and not smoking-exposed adolescents

^†^Geometric mean

^‡^Attention Deficit/Hyperactivity Disorder rating scale IV [13]: Total score (mother)

^§^Achenbach System of Empirically Based Assessment [12]: Mother report

Internalizing scale includes withdrawn, anxious/depressed, somatic complaints subscales.

Externalizing scale includes delinquent and aggressive behaviour subscales.

^¶^Autism Spectrum Screening Questionnaire [14]

**^$^**Adjusted for gender, birth weight, socioeconomic status, single parent, mothers’ use of alcohol, mothers’ age, and mothers’ mental health

General linear modelling (GLM)
